# Compact flexible multi-pass rotary delay line using spinning micro-machined mirrors

**DOI:** 10.1038/s41598-017-09576-5

**Published:** 2017-08-24

**Authors:** Roland Fleddermann, Woei Ming Lee, Keshu Huang, Geoff T. Campbell, Ping Koy Lam, Jong H. Chow

**Affiliations:** 1 Department of Quantum Science, Centre for Gravitational Physics, Research School of Physics and Engineering, The Australian National University, Canberra, ACT 2601 Australia; 2 Research School of Engineering, College of Engineering and Computer Science, The Australian National University, North Road, Canberra, ACT 2601 Australia; 3 Department of Quantum Science, Centre for Quantum Computation and Communication Technology, Research School of Physics and Engineering, The Australian National University, Canberra, ACT 2601 Australia

## Abstract

We propose a new method to extend the path length tunability of rotary delay-lines. This method was shown to achieve a duty cycle of >80% and repetition rates of over 40 kHz. The new method relies on a new multi-segmented micro-machined mirror and serial injection of a single reflection onto separate segments of this mirror. The tunability is provided by the relative positioning of each reflective point on the mirror segments. There are two distinct modes of operation: synchronous and asynchronous. By simply manipulating the spatial position of the returning paths over the respective mirror segments, we can switch between increasing the repetition rate (asynchronous mode) or the total delay path (synchronous mode). We experimentally demonstrated up to 8 m/s scans with repetition rates of up to 42.7 kHz. Furthermore, we present numerical simulations of 18 reflection points to illustrate possibility of achieving a scan speed of up to 80 m/s. Through intermediate combinations of synchronous and asynchronous operation modes with 4 or more passes, we also show that the system can simultaneously increase both repetition rate and scan depth.

## Introduction

Mirror-based optical delay lines deliver high optical efficiency and minimal dispersion for optical interferometry applications. Hence, these optical delay lines are easily applicable across a broad range of wavelengths in optical interferometry. These devices serve to provide accurate time-resolved measurements for spectroscopy^1^, communications^2^, coherence tomography^3–7^, and ultrafast ranging detection^8, 9^. It is worthwhile to note that delay lines using spectral shaping approaches produce high repetition rates of approximately two to four kHz while achieving tens of meters per second scan velocities. They operate by shaping individual wavelengths to achieve controlled delays using reflective gratings. However, this method suffers from relatively large power loss and is more sensitive to the incident wavelength range than traditional reflective delay lines due to the required diffraction efficiency.

For planar mirror-delay lines, the ability to increase the scan range can be generated through folding the delay path on large diameter mirrors^10–12^. However, any beam offset at the input needs to be carefully controlled so as to satisfy the geometry of the reflecting mirrors and to match a well-defined output point within a small volume^12^. The speed or travel range of the linear translator in the delay line is limited by the inertia of the mirror actuators.

To overcome some of the aforementioned limitations of other delay line techniques, rotary delay lines using machined mirrors were developed and used at near infrared wavelengths for optical coherence tomography. Lately, this type of delay line has also proven useful for high speed terahertz time domain spectroscopy^11^. While Liu and co-workers^4^ demonstrate a double-pass rotary delay line to increase path length, in their configuration and other similar approaches the repetition rate is still limited by the rotation rate of the motor and the number of mirror segments^4, 10^. In contrast, our new multi-pass rotary mirror scheme is shown to increase either the repetition rate or the scan range using the same number of mirror segments and rotation rate of the spinning disk. The main difference between our scheme and existing double pass configurations is that this new scheme allows one to tailor the overall optical path length difference (i.e. scan depth) to achieve either higher repetition rates at the same scan depth or increased scan depth at the same baseline repetition rate. This is all achieved through the synchronization of the relative position of each delay path over each pass. The technique also relies on the optimal use of a micro-machined mirror array where a large number of angled mirrors are tightly packed around the circumference of a small diameter disc. Hence, a single rotation of the disc can already yield a much higher baseline repetition rate than previous machined mirrors. Micro-machined optics offer low roughness surfaces and therefore exhibit a high level of optical reflectivity, which also contributes to a high overall optical efficiency of the system.

The basis of this scheme is to make full use of returning paths from separate reflecting surfaces of a single micro-machined mirror. The separation of successive passes maximizes coupling efficiency and path lengths and increases the scanning speed without increasing the size or the rotation rate of the disk. The new multi-pass approach extends the performance of existing rotary delay-lines and is directly applicable to many time-domain reflectometry/spectroscopy applications^9–11^. Variations of this technique may be applicable to other types of multi-pass mechanical delay lines.

In this paper, we first begin by illustrating the difference between existing double pass rotary delay lines and the new multi-pass approach. Next we describe how the multi-pass approach is implemented using standard fiber circulators and micro-machined mirrors. We then proceed to test the multi-pass delay line by integrating it into a time domain reflectometry system. The performance in scan range and scan repetition rate is demonstrated by imaging reflective samples, using both the synchronous and asynchronous mode of operation. These two modes differ in the way that each reflective point is positioned relative to each other on the mirror segments. Finally, we numerically extrapolate the performance of such a multi-pass delay to demonstrate the potential for higher scan repetition rates and depths, as well as mixed modes. Mixed modes allow for simultaneous increases in both scan repetition rate and scan depth.

**Figure 1:**
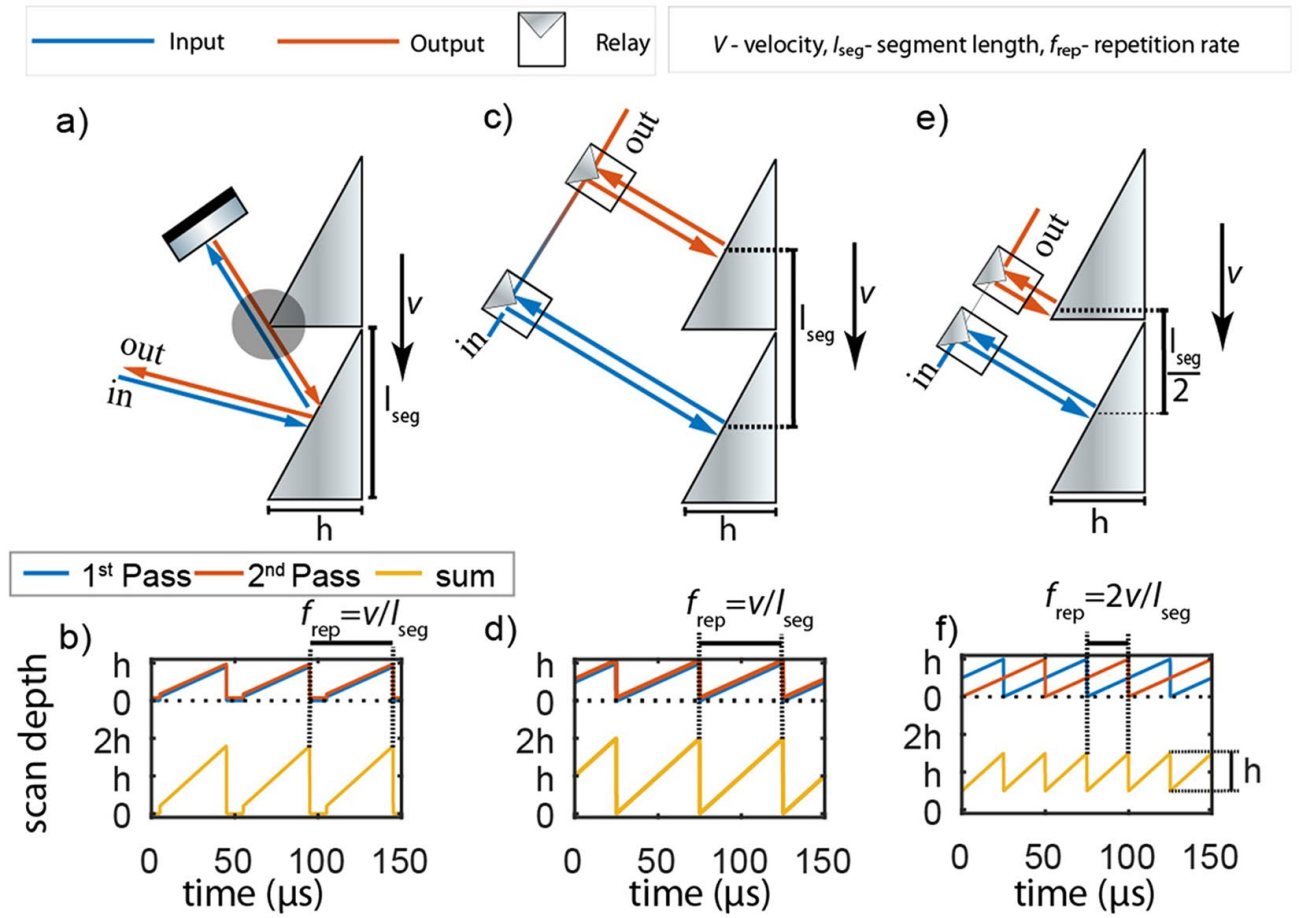
Comparison and operating principle of new multi-pass arrangement. (**a**) Previous rotary delay line double pass (**b**) achievable scan depth with mirror depth (*h*). (**c**) Double pass setup for the new multi-pass approach where the beam is reflected repeatedly from identical positions in 2 separate mirror reflectors (synchronous mode). (**d**) Achievable scan depth and scan rate using synchronous mode. (**e**) Double pass setup where the beam is reflected repeatedly from different position from separate mirror reflectors termed as asynchronous mode. (**f**) Achievable scan depth and scan repetition rate. A scan rate of 20 kHz is used as the base scan rate. *(note that the path length here is not drawn to scale)*.

Figure 1 shows the concept of previous double pass^4^ configurations alongside the new multi-pass approach. A rotating path with a linear velocity *v*, based on segments of angled mirrors with a given length (*l*_segment_) is assumed, which results in a scan repetition rate *f*_rep_ = *v*/*l*_segment_. Figure 1(a) illustrates the need for a small angle coupling if one is to apply a standard multi-pass approach to a rotary delay line. Figure 1(b) shows a time domain plot of the individual path length change for a single pass alongside with the achievable path length change in a double pass configuration. Here, we estimated the reduction in path length due to possible beam clipping resulting from the increased angle of incidence required for the double pass arrangement. Conversely, Fig. 1(c) shows the new multi-pass approach, where each pass is aligned orthogonally to each angled mirror segment for optimal light coupling. In this way, the scan depth is doubled without additional beam clipping. This delay line configuration exhibits optimal duty-cycle, coupling efficiency (discounting reflection losses) and resulting scan range, as illustrated in the time domain plot of path length change shown in Fig. 1(d). In addition, we also learnt that the multi-pass arrangement shown in Fig. 1(c) is just one variation of the multi-pass, termed as synchronous mode. In this mode, both reflection points fall on symmetrical points along the two separate mirrors segments, which leads to a maximum total path length change or scan range. Another variation of the multi-pass is shown in Fig. 1(e), termed here as asynchronous reflection mode. In this mode, the second reflection point is shifted by half a segment length, such that the total optical path length change exactly matches the single pass path length change while enhancing the scan repetition rate, as illustrated by the time series plot of the resulting path length change shown in Fig. 1(f). This subtle but important variation goes beyond a simple multi-pass approach, as the delay light can be tuned effectively between the two modes of operation by a small alignment change in one of the reflection paths.

## Multi-Pass Rotary Delay Line

**Figure 2:**
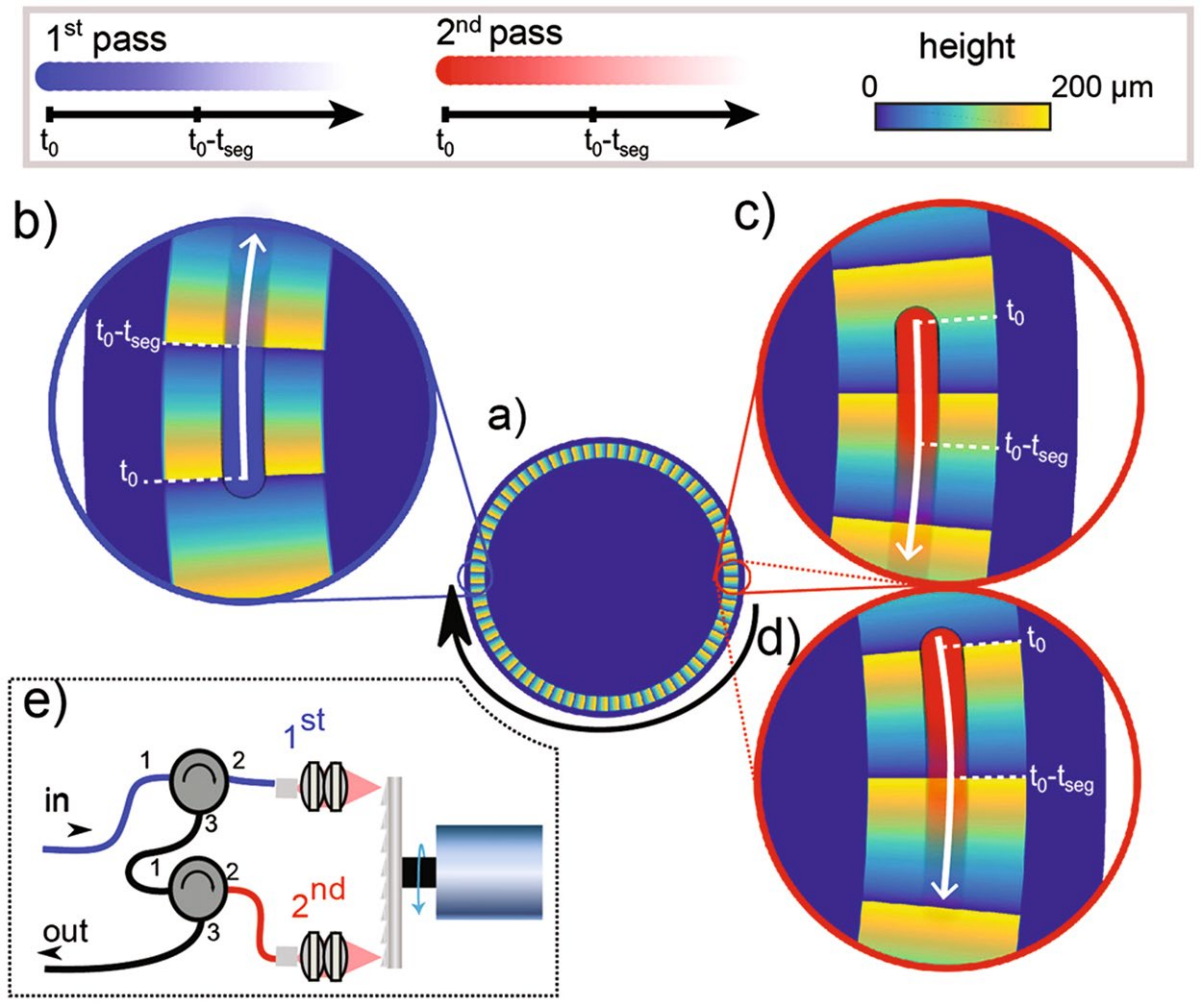
Schematic representation of reflection point positions and mirror shape for the implementation of a multi-pass system. The reflecting points are placed opposing each other over a disk with 69 mirror segments (**a**). The first reflection point is on the left side of the mirror (**b**). (**c**) and (**d**) show two options for the alignment of the second reflection point. The reflection points are shown for a period from an arbitrary starting time *t*_0_ to the time it takes for two segments to rotate under the beam (2 *t*_seg_) where *t*_seg_ = *l*_seg_/*v*.

In this section we describe the multi-pass as implemented with a micro-machined mirror. We used a fibre based reflectometry setup to demonstrate the capabilities of our delay-line. The core component of the delay line is a rotary mirror with a series of micro-machined mirrors arranged around the circumference of the disk in an annular fashion as illustrated in the false-color representation shown in Fig. 2(a), where the color represents the height above the surface of the mirror. To fabricate such high density of mirror elements (n = 69) over millimeter distances, we turn to nanolathe machining. The diamond turning tip on the nanolathe carves out individual mirror segment from a block of solid aluminum (for broadband reflectivity). The overall surface roughness of each mirror is sub-wavelengths (tens of nanometers) and the segments therefore exhibit high optical quality, resulting in high reflectivity and low scattering losses. Figure 2(b–d) shows the two modes of operations to achieve either longer depth scan or faster scan repetition rates as described in the previous section. Figure 2(b,c) show the first two reflecting positions for the asynchronous configuration in sequence. The fading red and blue circles show the reflection point of the first pass (blue) and the second successive pass (red), while the white and black arrows indicate the direction of rotation and the times at which the segments pass under the reflection points. In this configuration, the positions of the two reflection points relative to the mirror segments are different, they form the so-called asynchronous combination. As mentioned above, this configuration results in a doubled scan repetition rate while the scan depth remains equal to the absolute height of the individual mirror segments (as is the case in a single pass configuration). By slightly displacing the second reflected path from the asymmetric arrangement shown in Fig. 2(c) to a position where the relative reflection points are aligned onto the same position at each mirror segment, the configuration can be altered to yield the synchronous combination. In this case, the reflection point is shifted by half the length of one segment upon the second reflection off the mirror segments, as illustrated in Fig. 2(d). This synchronous alignment achieves an increase in the scan depth while the same scan repetition rate of a single pass arrangement is maintained.

Finally, a side view schematic of the multi-pass arrangement using fiber optic circulators is illustrated in Fig. 2(e). While we chose to use fiber optics to implement the double-pass beam routing because it is directly compatible with fiber based optical reflectometry, the same result could have been achieved with free space optical components (polarization beam splitters and wave plates).

**Figure 3:**
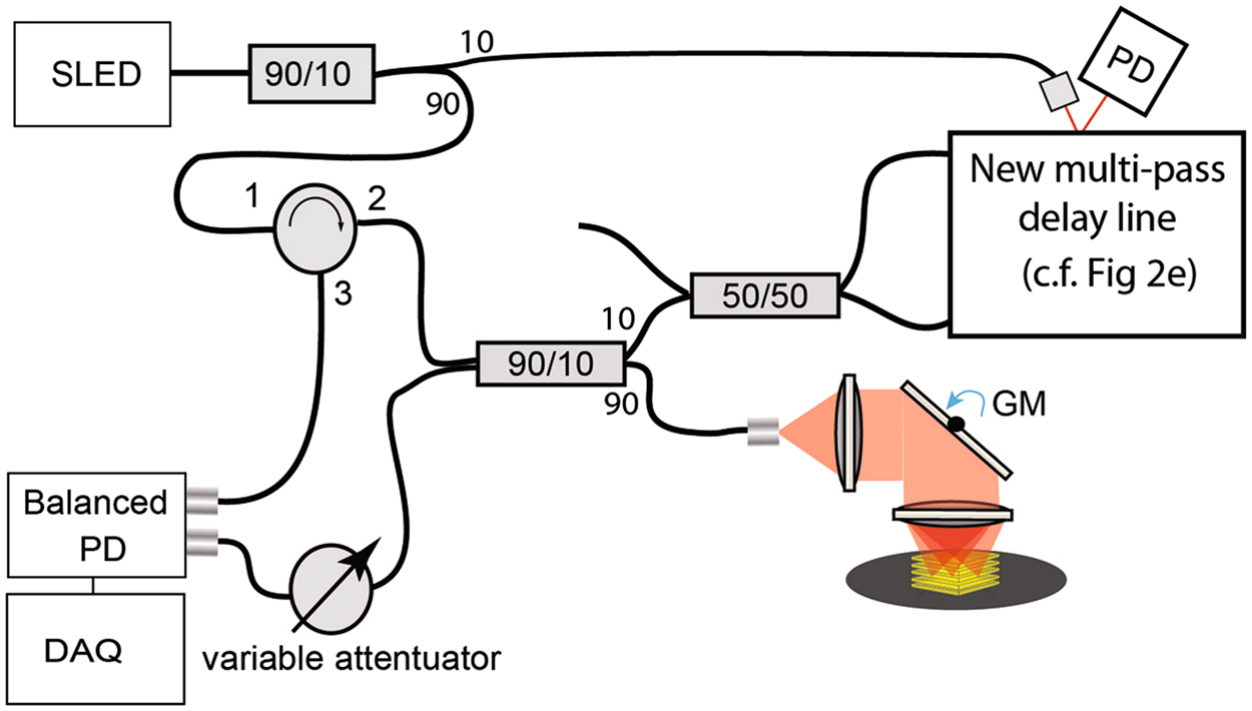
Schematic of the time-domain reflectometry setup with multi-pass delay line, with the spinning delay line incorporated in the reference arm. A thin reflective sample was placed on a speaker in the sample arm, driven with a sine wave at 40 Hz. The reflected signals from the reference and sample arm were combined by a 90:10 coupler into a variable attenuator and then a balanced photodetector. The interference signal was digitized and filtered.

The extended experimental setup of the fiber-reflectometry demonstration experiment with the multi-pass delay line inserted into the reference path is shown in Fig. 3. As light source we used a fiber coupled SLED (1294 ± 42.5 nm) connected to a 90/10 fiber coupler; 90% of the light was directed onto an initial circulator used to facilitate balanced detection and entered another 90/10 splitter. This second 90/10 splitter was used to distribute the light into the reference arm and sample arm. In the reference arm, a 50/50 splitter was used to launch the initial beam into the first circulator and onto the first facet of the structured mirror. The returning beam (output 3 of the first circulator) was fed into a second optical circulator and projected onto a second mirror facet on the opposite side of the mirror. After undergoing these two reflections the beam then re-entered the 50:50 coupler to be directed back to the interference coupler and be combined with the reflected signal from the sample arm. The resulting 50 percent (3 dB) loss in the reference arm from using this beam routing solution was acceptable for the purpose of our demonstration experiment, but can be circumvented by using more sophisticated optical configurations. The eventual interference signal was detected using a balanced photodetector (New Focus 1817-FC). The interference signal was then digitized and filtered using an analogue to digital converter (MAX 1427 15-bit 80 Msps ADC) and a National Instruments FPGA card (PXI 7854 R) for real-time data acquisition and processing.

Additionally, a two-axis galvanometer mirror was placed in the sample arm to provide raster scanning for spatially resolved reflectance measurement. The raster scanning motion was synchronized to the motor rotation rate and the resulting delay scans using the 10 percent output of the first coupler, which was directed onto the side of the mirror. The resulting reflection was detected using a second detector (Newport 1811). While this detector has a bandwidth of 125 MHz, this is by no means required and a low-bandwidth switchable gain detector could have been used just as easily as a trigger signal for data acquisition and galvanometer scan signal generation.

## Results

**Figure 4:**
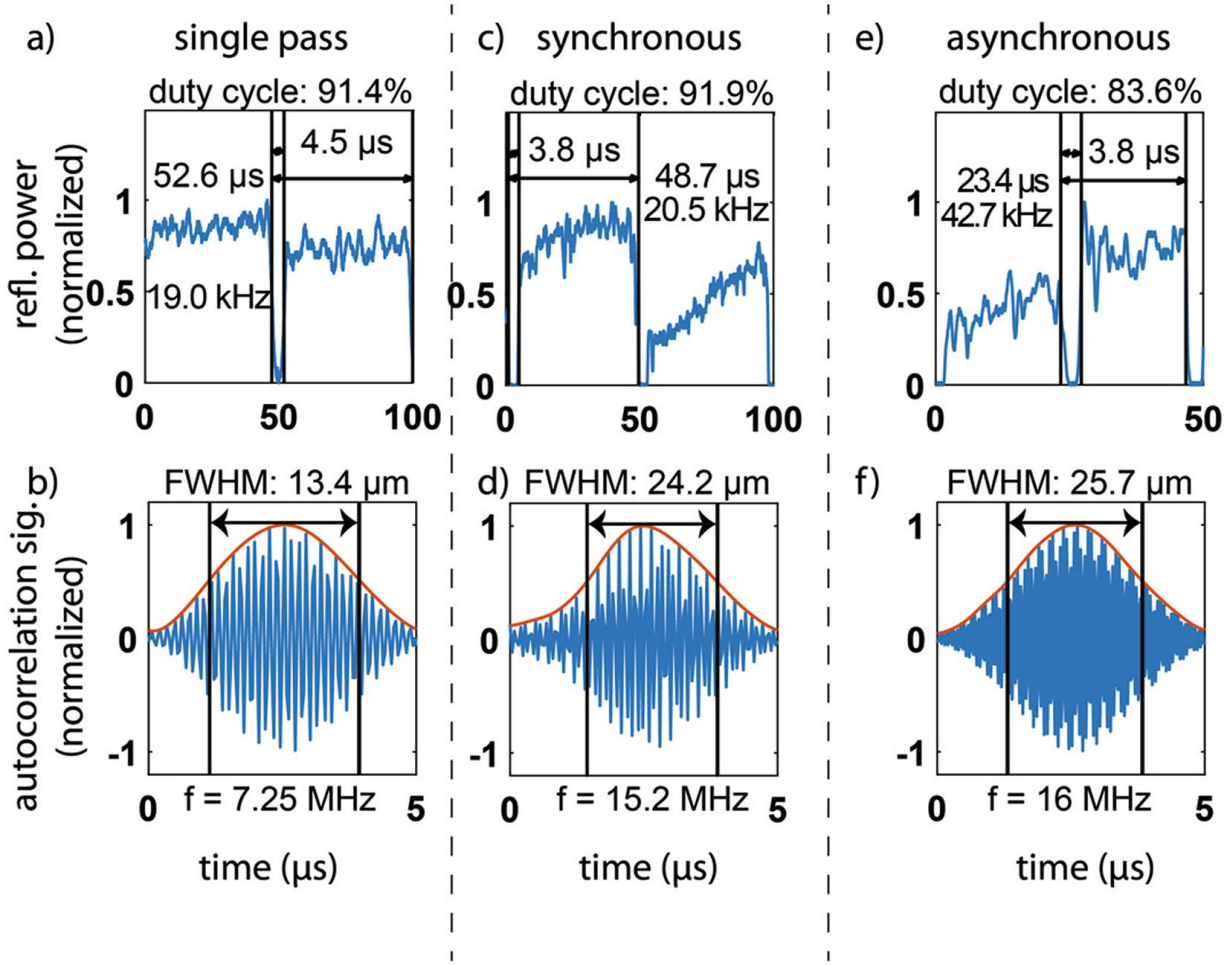
Time domain plots of the reflected power ((**a**), (**c**), (**e**)) for assessment of duty cycle and autocorrelation signal ((**b**), (**d**) and (**f**)) from axial scans. (**a**) and (**b**) shows single pass, (**c**) and (**d**) shows the synchronous approach and (**e**) and (**f**) details the asynchronous approach. The duty cycle and repetition rate measurements shown are based on the amplitude of the back reflected signal while the resolvable path length difference and Doppler frequency are measured from the autocorrelation function.

**Figure 5:**
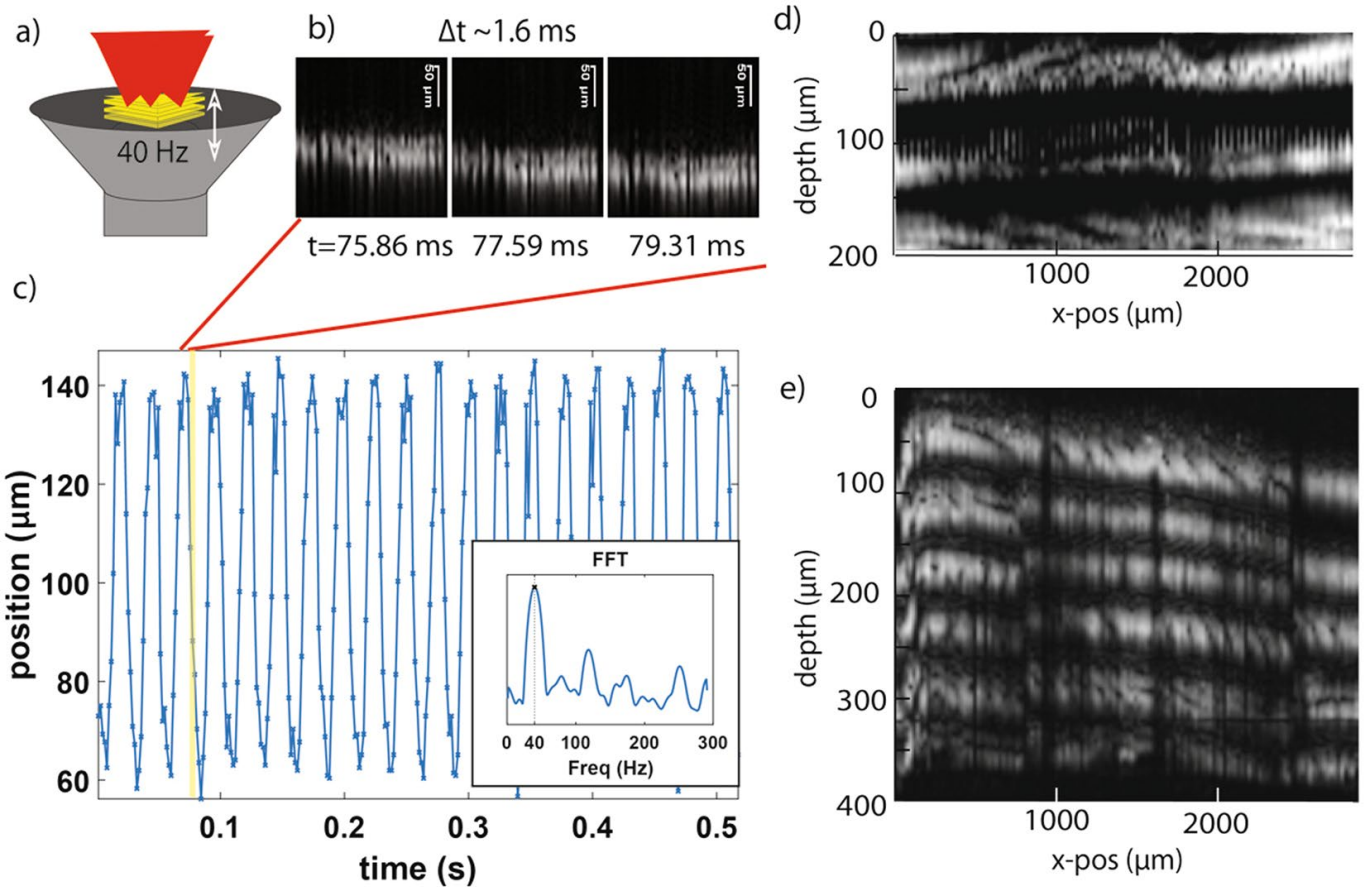
Experimental demonstration of the achievable scan repetition rate and scan depth using a vibrating film on a speaker and a stack of thin transparent films. (**a**) Illustration of the stacks of reflective film mounted onto an acoustic speaker in the sample arm of our reflectometry setup. (**b**) Three cross-sectional images (B-scans) of the reflected amplitude (grayscale intensity representation) over a scan depth of 200 µm (scale bar 50 µm) obtained at a frame rate of 580 Hz (68 × 476 format) to capture millisecond action of the reflective film vibrating at 40 Hz. (**c**) Plot of the full oscillatory motion of the film’s center position over multiple cycles (inset shows the FFT spectrum). Cross sectional images of a stack of thin films for scan range assessment using reflectometry with synchronous beam position (**d**) and asynchronous beam position (**e**).

The experimental data acquired using the optical fiber reflectometry setup to investigate and demonstrate several aspects of our new multi-pass technique is presented in Figs 4 and 5. In a first experiment we aimed to demonstrate the achieved scan repetition rate and duty-cycle. To this end, we monitored the reflected power of the delay line as well as the resulting interference signal. We obtained the reflected power signal by disconnecting one of the balanced detector’s inputs and blocking the sample arm. This measurement shows the duty-cycle obtained in either of the three configurations (single-pass, synchronous and asynchronous double pass). These time domain signals are presented in Fig. 4(a,c and e), respectively. Using glass coverslips in the reference arm, we were able to obtain interference signals to determine the quality of the autocorrelation signal that is typically used for time resolved imaging or spectroscopy. These time domain signals are shown in Fig. 4(b,d and f), for single-pass, synchronous and asynchronous double pass, respectively. We used a rotating disk with 69 micro-machined mirror segments spinning at a frequency of 300 Hz (18,000 rpm).

To obtain information about the duty-cycle of each configuration, we used the reflected signal. The duty-cycle is simply the ratio of the time interval where the reflected power is above half of the maximum $({{\bf{t}}}_{ > {\bf{50}} \% })$ divided by the total cycle time $\,{{\boldsymbol{T}}}_{{\rm{rep}}}$. To represent this number as a percentage (as is customary), the ratio is multiplied by 100, i.e. duty-cylce ${\boldsymbol{D}}=({{\bf{t}}}_{ > {\bf{50}}{\boldsymbol{ \% }}}/{{\bf{T}}}_{{\rm{rep}}})\times {\bf{100}}$. Similarly, we use the information from the same measurement to determine the repetition rate, finding the period after which the signal repeats $({{\boldsymbol{T}}}_{{\rm{rep}}})$. This results in a repetition rate or frequency ${{\boldsymbol{f}}}_{{\rm{rep}}}={\bf{1}}/{{\boldsymbol{T}}}_{{\rm{rep}}}$. To infer the scan range we use the Doppler frequency obtained from the observed autocorrelation signal. The scan range can be inferred from the observed Doppler frequency by dividing it by the repetition rate and multiplying it by the half the wavelength of light used. When the duty-cycle is less than one, it also needs to be taken into account by multiplying the scan range by it. Thus, the total scan range is found to be ${{\boldsymbol{d}}}_{{\rm{scan}}}=D{\boldsymbol{\lambda }}/{\bf{2}}{{\boldsymbol{f}}}_{{\rm{Doppler}}}/{{\boldsymbol{f}}}_{{\rm{rep}}}$.

Using just single pass (removing the second pass) on a single mirror segment we obtained a base repetition rate $\,{{\boldsymbol{f}}}_{{\rm{rep}}}$ of 19.0 kHz (52.6 µs) with a duty cycle of 91.4% as demonstrated by the reflected amplitude signal presented in Fig. 4(a). Figure 4(b) shows the corresponding auto-correlation signal with a resolvable path length difference of 13.5 µm over 2.8 µs (in air), which corresponds to a Doppler frequency of about 7.25 MHz and an overall scan depth $({{\boldsymbol{d}}}_{{\rm{scan}}})$ of 224 µm, in agreement with the depth of the individual mirror segments.

For synchronous mode, the repetition rate remains nearly unchanged at 20.5 kHz and the duty cycle is also comparable at a value of 91.9%, as demonstrated by the reflected power signal shown in Fig. 4(c). These values are almost identical to the single-pass. However, in this arrangement we find that the scan range is doubled. This is demonstrated by the autocorrelation signal shown in Fig. 4(d). We find that the path length resolution is now 24.2 µm over 2.4 µs at a Doppler frequency of 15.2 MHz, which combined with the repetition rate leads to a scan range of 438 µm.

Figure 4(e) shows the reflected power in asynchronous mode. The repetition rate is now found to be 42.7 kHz (23.4 µs), which is twice that of the base scan speed. The duty cycle is only slightly reduced to 83.6%. The corresponding measured interference fringes shown in Fig. 4(f) indicate a slightly reduced path length resolution of 25.8 µm over 2.5 µs, corresponding to a Doppler frequency of 16 MHz and a scan range of 202 µm which is slightly reduced compared to the single pass due to the reduced duty-cycle.

To further experimentally verify the increase in scan speed and scan range using the reflectometry system, we conducted another series of experiments with the goal to demonstrate the potential high imaging speed using reflective films oscillating over an acoustic speaker and stacked on top of each other. In a first experiment, we used a single reflective film placed on an audio speaker, driven by a sine wave with a frequency of approximately 40 Hz, as shown in Fig. 5(a). Using the asynchronous configuration, we were able to achieve a scan repetition rate of 42 kHz and directly capture this oscillatory motion over multiple cycles. We captured axial cross-section images with an elongated aspect ratio (68 × 476 scan points) that provided 580 images per second, using a galvanometer mounted mirror to scan the beam across a line at a frequency of 580 Hz, which is within the bandwidth of the galvanometer. This enabled us to capture 1.6 millisecond dynamics of the vibrating film with minimal imaging artifacts (close inspection of the cross-sectional images reveals that the edges of the image are found to be at slightly different depths, consistent with the finite amount of movement during the 1.6 ms scan time). Figure 5(b) shows three false-color representation cross-section snapshots of the reflected signal, scaled for easier visual inspection. We tracked the center position of the film in these images over 600 frames (about 1 s); shown in Fig. 5(c) is the time dependent z (depth) position of the film’s center.

To verify the scan range in both configurations, we captured cross-sectional images of a stack of multiple reflecting surfaces over 200 µm in depth as shown in Fig. 5(d). To increase the depth scan range, we reverted to the synchronous positioning which then captured a longer range of around 400 µm as shown in Fig. 5(e). The thickness of the individual film layers is 75 µm.

### Multiple-passes (>4) and mixed synchronous/asynchronous modes

**Figure 6:**
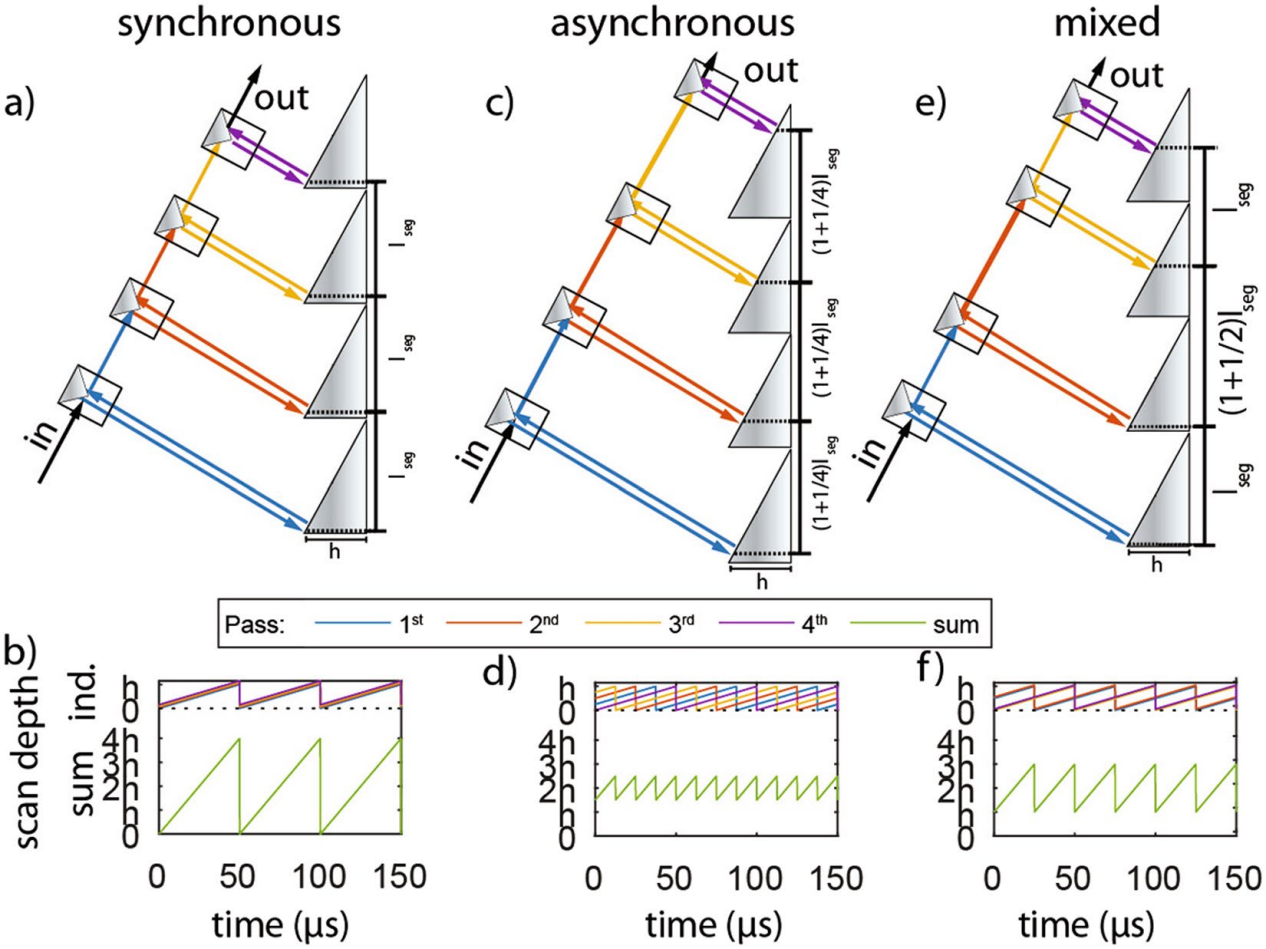
Additional scan repetition rate/scan depth combinations achievable with at least four passes. Illustrated for 4 passes we show the synchronous quadruple pass in sub-Figure (**a**,**b**), showing the configuration and time dependent delay respectively, (**c**,**d**) showing the same graphs for asynchronous configuration and finally (**e**,**f**) showing the same for the mixed case. We again assume a base scan repetition rate of 20 kHz.

Section 2 primarily discusses the synchronous and asynchronous configurations using just two passes. However, the micromachined multi-facet mirror can easily accommodate many more passes (>3). In doing so, we can start to mix the different degrees of synchronization through the phase of individual passes. Figure 6(a) shows synchronous combination of four passes on each corresponding mirror facet, which increases the scan depth by a factor of four at the same repetition rate, as indicated by the time series plot of the individual delays and the resulting combined delay shown in Fig. 6(b). On the other hand, the asynchronously arranged passes shown in Fig. 6(c) result in an increase in speed by a factor of four while the scan depth remains at the height of each mirror facet. Again, this is accompanied by a plot of the individual delays and their sum indicating the resulting increase in scan repetition rate while the scan range remains unchanged compared to a single pass. Additionally, using four passes we can achieve a new interesting combination which allows us to both increase the repetition rate as well as the scan depth. This is done by aligning two passes to be synchronously matched while adjusting the position of the remaining two passes to be asynchronous with the first two. This combination, as illustrated in Fig. 6(e), leads to an increase of both the scan speed and depth by a factor of two simultaneously. We plotted the expected delays in Fig. 6(f) for such a mixed configuration. Generally speaking, any integer divisor of the number of passes can be used to split the passes between synchronous and asynchronous configurations, leading to 6 possible combinations for 12 passes (1/12, 2/6, 3/4, 4/3, 6/2 and 12/1) and 18 passes (1/18, 2/9, 3/6, 6/3, 9/2 and 18/1).

**Figure 7:**
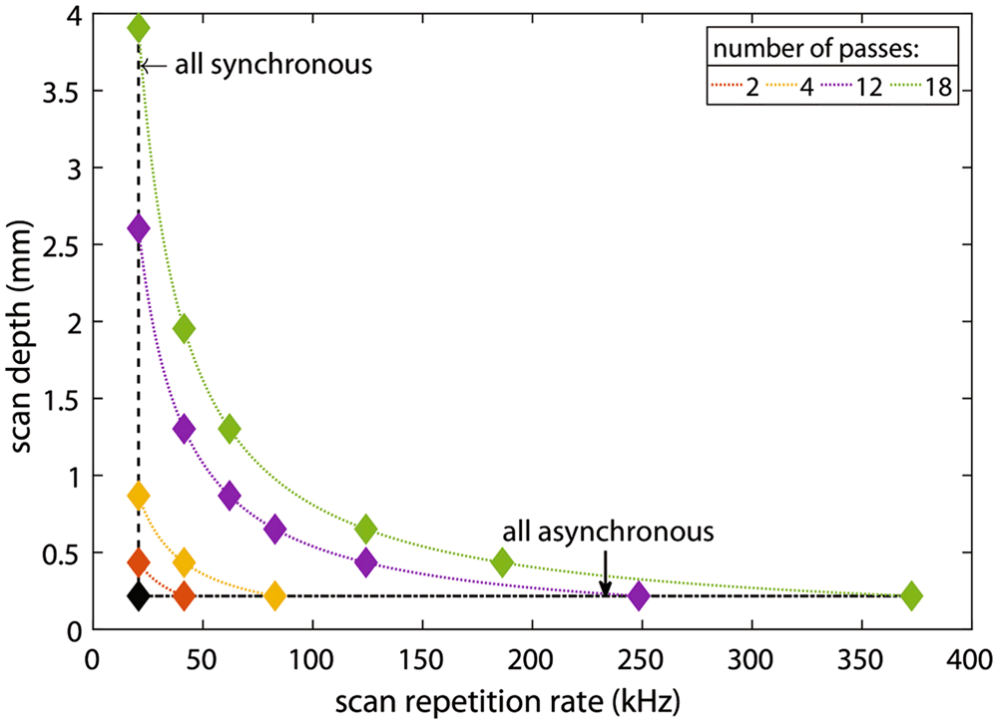
Different scan repetition rates and scan depths achievable with increasing number of passes. The black diamond highlights the actual configuration chosen (69 segments with a slope of about 5 degree, leading to a segment height of about 200 µm). Additionally, all achievable scan rate/depth combinations using a multi-pass approach with 2,4,12 and 18 passes, indicated by orange, yellow, purple and green diamonds, respectively are highlighted. The leftmost diamonds indicate synchronous configurations with constant repetition rate and increasing scan depth, while the rightmost ones correspond to (fully) asynchronous configurations with increasing repetition rate but constant scan depth. In between are the mixed cases, as illustrated for 4 passes in the previous Figure for a mixed case of 2 synchronous and 2 asynchronous passes.

Based on the large degree of freedom offered by our multi-pass scheme, we also further examined and compared the performance based on higher numbers of reflecting points/passes. We based our calculation on the current 2-inch diameter disk with 69 angled mirror segments each with a height of 200 µm. Figure 7 explores possible repetition rates and scan depths achievable using an extended multi-pass approach. The black diamond highlights the design parameters we chose for our micro-mirror array of 69 mirror segments and 5 degree slope which results in a scan depth of about 200 µm and a scan repetition rate of about 20 kHz where we have verified the performance with our experimental setup. The plot shows the resulting scan depth and repetition range for a selection of passes ranging from 2 to 18 passes. Based on the combination of individual passes, we calculated that the system is capable of yielding scan depths of up to 3.6 mm, with 18 passes arranged synchronously, or repetition rates of up to 0.36 MHz, with 18 passes arranged asynchronously. In both cases, the equivalent scan speeds can reach 200 m/s. All this is achieved without changing the basic micromachined mirror design but only utilizing multiple-reflections/passes off the same mirror. Through this simple but powerful arrangement of multiple passes on a spinning micromachined mirror, it has surpassed any previous practical limitations of traditional rotating delay lines. Using this approach we can simplify the enhancement of the multiple passes by associating the maximum achievable scan depth to be simply the product of the number of passes (N) and the single pass scan depth, while the maximum achievable scan speed is the product of the number of passes (each arranged with a phase delay of 360°/N) and the base scan speed of the delay line. In either case, the total scan speed measured in meters per second is increased by a factor of N.

## Conclusion

We presented a new multi-pass configuration for optical delay lines is expands traditional single reflecting path over a rotary delay line and addresses the limited flexibility in extending scan depth or scan rate. By using our new multi-pass technique, the repetition rate or scan depth can be increased independently. We demonstrated a scan rate of 42 kHz, which exceeds the scan rate of previous rotating scanners by more than an order of magnitude. We also presented simulations that illustrate that further increases in scan range and repetition rate could be achieved using a higher number of passes. A remaining challenge for this increase in the number of folded delays is to maintain efficient coupling of the reflected light back into the fiber. The physical distance between each collimator and the rotating disk needs to be sufficiently spaced out to ensure good power coupling. We therefore plan to address this in future experiments by constructing a customized GRIN fibre-coupler array that has the potential to achieve either sub-MHz frequency or tens of millimeter scan range.
